# Natural products in traditional Chinese medicine for renal fibrosis: a comprehensive review

**DOI:** 10.3389/fphar.2025.1560567

**Published:** 2025-04-16

**Authors:** Qianqian Zhao, Meihua Jin, Qiang Zhao, Zhimei Wang, Chun Zhao, Xiaocong Xue, Xikai Qiao, Peng Qu, Donghe Han, Ran Tao

**Affiliations:** ^1^ Department of Anatomy, Medical College, Dalian University, Dalian, Liaoning, China; ^2^ Department of Immunology, Medical College, Dalian University, Dalian, Liaoning, China

**Keywords:** renal fibrosis, epithelial-mesenchymal transition, natural products, traditional Chinese medicine, inflammation, oxidative stress, TGF-β

## Abstract

Renal fibrosis represents the terminal pathological manifestation of most chronic kidney diseases, driving progressive loss of renal function. Natural products have emerged as promising therapeutic agents for preventing and ameliorating renal fibrosis due to their multi-target efficacy and favorable safety profiles. In this review, we conducted a comprehensive literature search on PubMed using the keywords “natural product” and “renal fibrosis” from 2004 to 2025, identifying 704 relevant articles. We systematically categorize and discuss the biological effects of key natural products and formulations with antifibrotic potential, focusing on five major classes: glycosides, flavonoids, phenolic compounds, anthraquinones, and terpenoids. Representative compounds from each category are highlighted for their mechanisms of action, including modulation of oxidative stress, inflammation, autophagy, and fibrosis signaling pathways. This review aims to provide a theoretical foundation for the development of natural product-based therapies to combat renal fibrosis, offering insights into their therapeutic potential and future research directions.

## 1 Introduction

**FIGURE 1:**
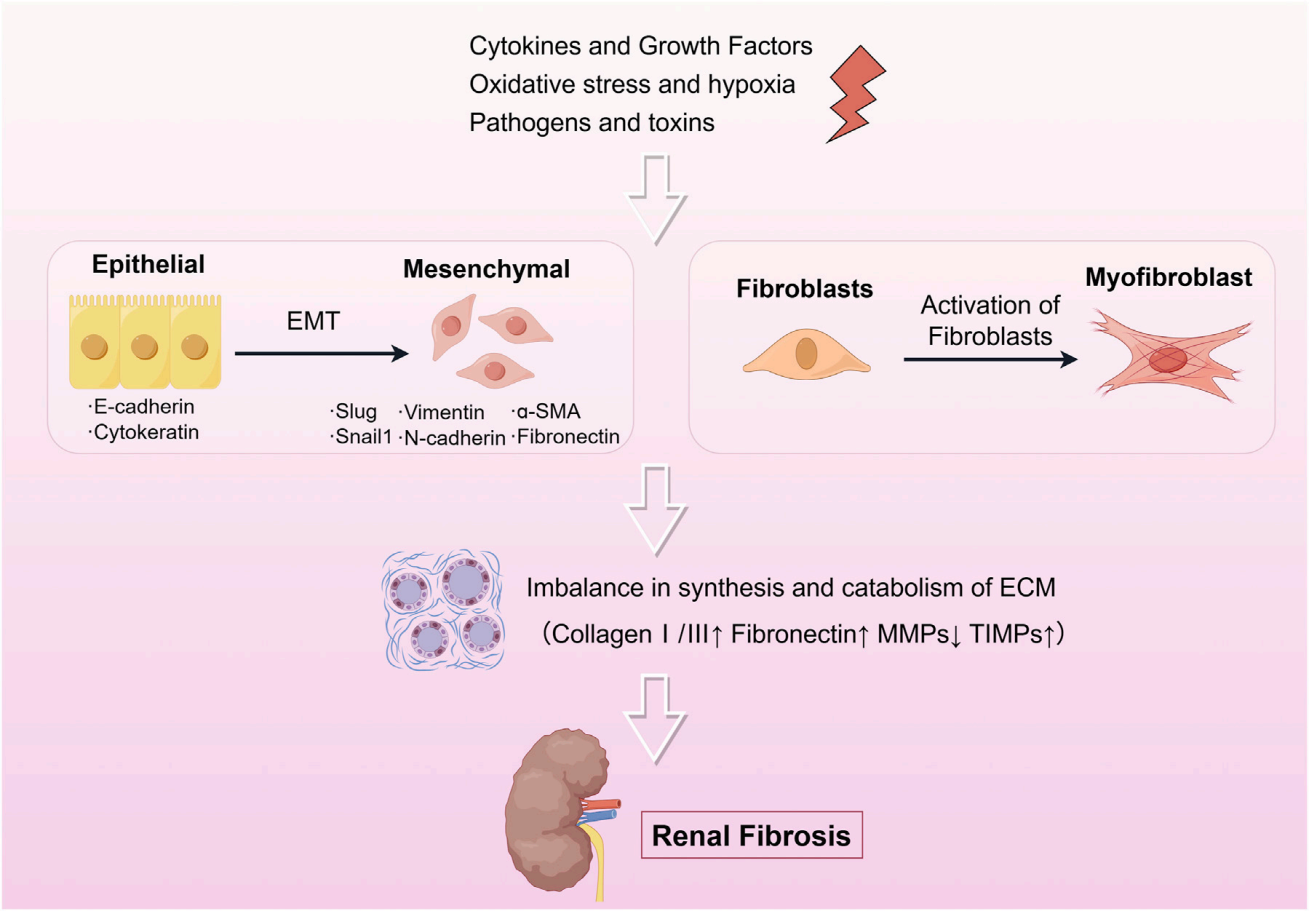
The pathophysiology of renal fibrosis. Renal fibrosis is a complex pathological process that occurs mainly by activation of EMT and fibroblasts under various factors such as cytokines, growth factors, oxidative stress, hypoxia, pathogens, and viruses, which in turn leads to an imbalance between synthesis and catabolism of the ECM leading to fibrosis. The drawings in this review were made by Figdraw.

The rising global prevalence of hypertension, diabetes, and obesity has precipitated a parallel increase in kidney disease incidence, imposing a significant public health burden (Jing et al., 2024). Renal fibrosis, the terminal pathological manifestation of most chronic kidney diseases (CKD), is a multifactorial process driven by extracellular matrix (ECM) dysregulation, fibroblast-to-myofibroblast transdifferentiation, immune cell infiltration, and tubular epithelial-mesenchymal transition (EMT)(Figure 1). EMT, a pivotal mechanism in fibrogenesis, involves the phenotypic transformation of polarized epithelial cells into motile mesenchymal cells, marked by loss of apical-basal polarity, dissolution of intercellular adhesions, and acquisition of migratory and invasive properties. This process is central to embryogenesis, wound healing, fibrotic disorders, and metastatic progression (Li et al., 2024; Sugyeong et al., 2022; Lili and Shougang, 2020). A hallmark of EMT is cadherin switching, characterized by transcriptional repression of E-cadherin—a calcium-dependent adhesion molecule critical for maintaining epithelial integrity through β-catenin binding—and concomitant upregulation of N-cadherin. E-cadherin depletion destabilizes adherens junctions, facilitating cytoskeletal reorganization and mesenchymal marker expression, including α-smooth muscle actin (α-SMA). Keratin, another conserved epithelial marker, inversely correlates with fibrotic progression, as evidenced by its downregulation in EMT-driven renal fibrosis (V et al., 2024; Brittany et al., 2024). Mechanistically, transforming growth factor-β1 (TGF-β1) and its downstream effector Snail orchestrate EMT by suppressing epithelial markers (e.g., cytokeratin) and inducing mesenchymal markers (e.g., α-SMA), with their expression levels positively correlating with renal interstitial fibrosis severity (Xiao-Yi et al., 2016). These insights underscore the therapeutic potential of targeting EMT-associated pathways to mitigate renal fibrosis, emphasizing the need for precise modulation of cadherin dynamics and TGF-β signaling pathways.

**FIGURE 2:**
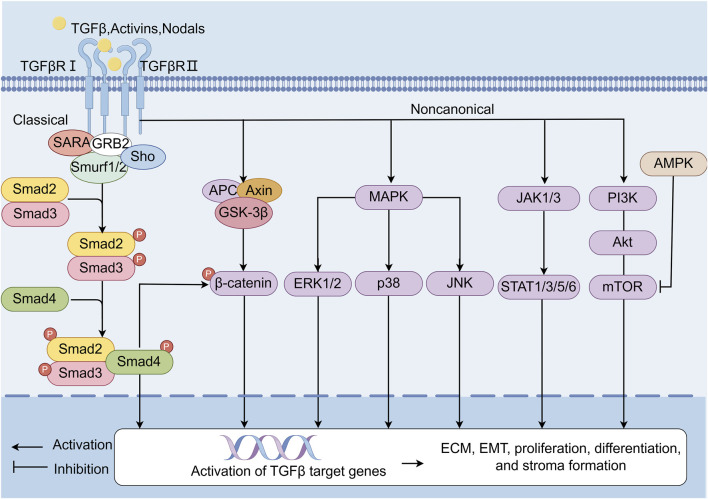
TGF-β/Smads signaling pathway. TGF-β/Smads is one of the classic pathways of fibrosis. The canonical pathway refers to the TGF-β/Smads signaling pathway. Ligands such as TGF-β, activins, and nodal bind complexes of transmembrane receptor types I and II on the cell surface, p-Smad2, and p-Smad3. Then, these ligands form a complex with Smad4. The activated Smad complex is transferred to the nucleus and binds to site-specific recognition sequences in the promoter regions of target genes to directly regulate transcription. In noncanonical pathways, the TGF-β receptor complex signals through other factors—such as β-catenin, MAPK (ERK1/2, p38 and JNK), JAK1/3-STAT1/3/5/6, and the PI3K-AKT-mTOR signaling pathway—which are indirectly activated by TGF-β target genes and involved in EMT, proliferation, differentiation, and stroma formation. In addition, AMPK inhibits mTOR, thereby affecting the PI3K-AKT pathway, which is involved in the regulation of fibrosis.

The TGF-β/Smad signaling pathway represents a canonical mechanism underlying fibrotic progression (Figure 2) (Xinyue et al., 2024; Xiao-Ming et al., 2016; Dandan et al., 2022; He-He et al., 2018; Jun Ho and Joan, 2022; Nikolaos, 2020; Erine et al., 2021). This pathway bifurcates into canonical and noncanonical branches. The canonical TGF-β/Smad cascade initiates when ligands such as TGF-β, activins, or nodal bind to transmembrane type I and II receptor complexes, triggering phosphorylation of Smad2/3. Phosphorylated Smad2/3 subsequently associates with Smad4, forming a heteromeric complex that translocates to the nucleus to regulate transcription of fibrogenic target genes. In contrast, noncanonical TGF-β signaling engages alternative effectors-including β-catenin, Mitogen-activated protein kinase (MAPK) (ERK1/2, p38 and JNK), JAK-STAT, and PI3K-AKT-mTOR pathways-to indirectly modulate EMT, cellular proliferation, and stromal remodeling (Xingmei et al., 2024). In addition, AMP-activated protein kinase (AMPK) inhibits mTOR, thereby affecting the PI3K-AKT pathway, which is involved in the regulation of fibrosis (Hui-Yun et al., 2020). Elevated TGF-β1 expression in fibrotic kidneys underscores its pivotal role in driving ECM deposition and tubular EMT, hallmark features of renal fibrosis (Dandan et al., 2022; Constantinos et al., 2019). Consequently, therapeutic strategies targeting TGF-β1 inhibition form a cornerstone of antifibrotic interventions, including those derived from natural products.

**FIGURE 3:**
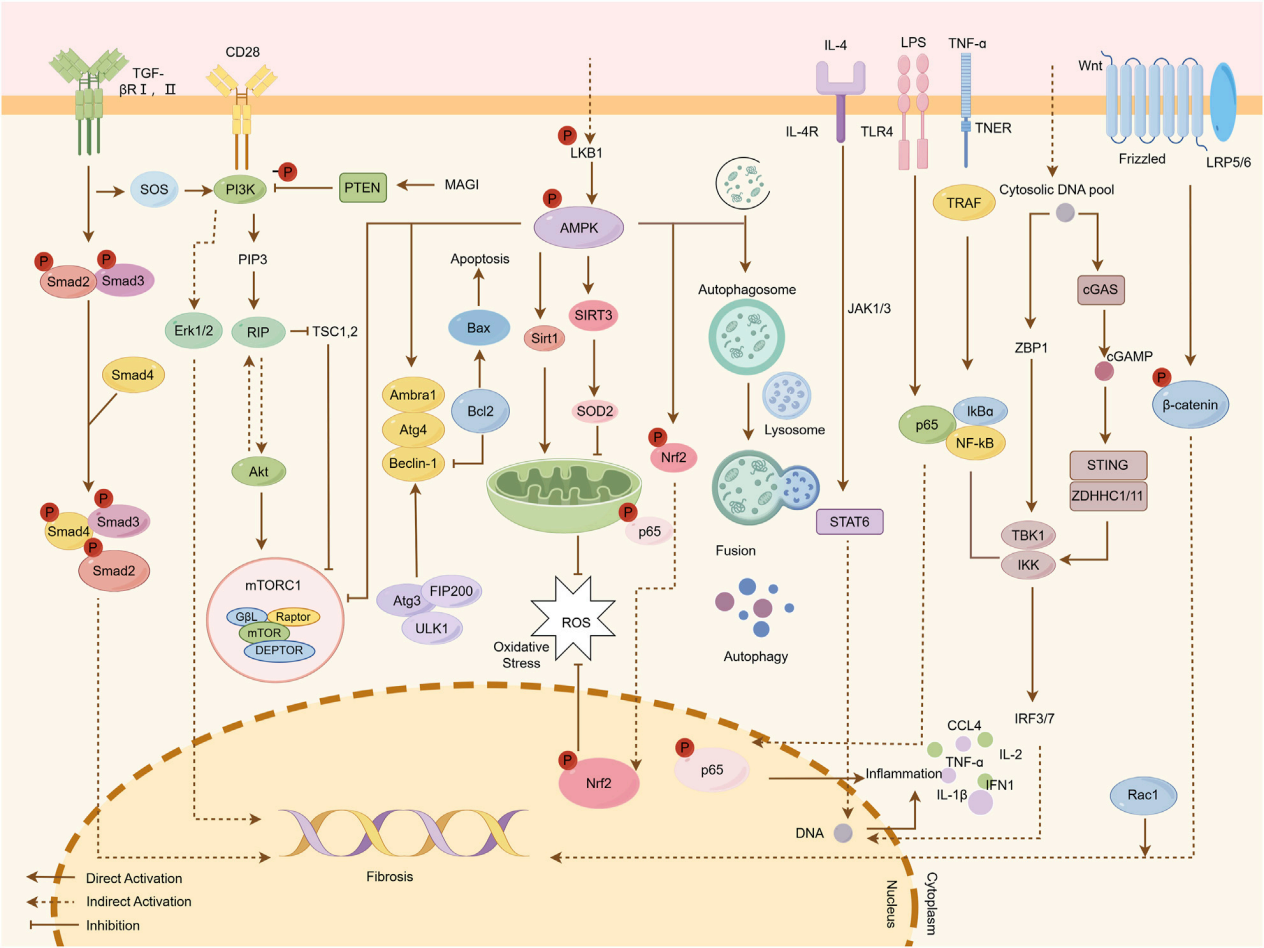
Mechanisms by which natural products ameliorate renal fibrosis. Fibrotic progression is driven by the interplay of multiple signaling pathways. The TGF-β/Smad pathway is initiated when TGF-β binds to TβRI and TβRII receptors, leading to the phosphorylation of Smad2/3. These molecules form a complex with Smad4, which translocates to the nucleus to regulate fibrogenic gene expression. The PI3K/Akt/mTOR pathway is activated by upstream signals such as CD28, resulting in the phosphorylation of PIP3 and Akt, which subsequently activate mTOR and Erk1/2, promoting fibrosis. The AMPK pathway mitigates fibrosis by enhancing autophagy, regulating oxidative stress through Sirt1 and SOD2 activation, and promoting apoptosis via Beclin-1. In the JAK/STAT6 pathway, IL-4 binding to cell surface receptors activates JAK1/3 kinases, leading to STAT6 phosphorylation and nuclear translocation, where it regulates target gene transcription. The cGAS/STING pathway mediates DNA-induced immune responses; cGAS recognizes cytosolic DNA and synthesizes cGAMP, which activates STING and downstream IRF3/7 and NF-κB pathways, inducing type I interferons (IFN-I) and pro-inflammatory cytokines (e.g., CCL4, TNF-α, IL-1β). Additionally, the Wnt/β-catenin pathway, when activated, stabilizes β-catenin, allowing its nuclear translocation to regulate genes involved in fibrosis. Natural products attenuate renal fibrosis by targeting these pathways, modulating inflammation, oxidative stress, apoptosis, and autophagy.

Traditional Chinese Medicine (TCM) has emerged as a promising adjunctive therapy for renal fibrosis, leveraging its pleiotropic mechanisms to restore biological homeostasis. Key pathological drivers—oxidative stress, inflammation, and autophagy-apoptosis dysregulation-are intricately interconnected in fibrogenesis. Firstly, excessive reactive oxygen species (ROS) generation activates proinflammatory cascades, exacerbating ECM deposition and tubular injury. Conversely, inflammatory mediators such as tumor necrosis factor alpha (TNF-α) and interleukin (IL)-6 amplify ROS production via NADPH oxidase activation, establishing a self-perpetuating cycle (Mengqi et al., 2024; Samar et al., 2023; Xiao-Jun et al., 2024; Jiawei et al., 2020). TCM counteracts this axis by activating AMPK, enhancing energy homeostasis and reducing lipid peroxidation (Li et al., 2015; Nan et al., 2016). Secondly, oxidative-inflammatory axis further disrupts autophagy, impairing clearance of damaged organelles and proteins, which perpetuates cellular dysfunction and fibrosis (Jun-Hao et al., 2022; Rong et al., 2022; Feng Y. et al., 2024; Azam et al., 2018; Maria et al., 2018), while TCM restores autophagic flu and mitigates oxidative damage by inhibition of PI3K/AKT/mTOR (Virginia et al., 2019; Changlong et al., 2021). Cellular stress responses are governed by a delicate equilibrium between autophagy and apoptosis. Under mild stress, autophagy promotes cell survival by degrading dysfunctional components, whereas severe injury shifts the balance toward apoptosis. Dysregulated autophagy leads to ROS accumulation and myofibroblast activation, while excessive apoptosis releases proinflammatory cytokines, fueling fibroblast proliferation and ECM deposition (Wafa Ali et al., 2025; Y et al., 1997; Xing-Chen et al., 2019; Siwei et al., 2021; Hui Z. et al., 2022). TCM modulates this balance by downregulating cyclic GMP-AMP synthase (cGAS)/stimulator of interferon genes (STING) signaling, thereby limiting DNA damage-induced inflammation (Meifang et al., 2024; Kathy et al., 2016). Moreover, TCM exerts influence on JAK/STAT6 signaling pathways as well as Wnt/β-Catenin to inhibit inflammation and tubular dedifferentiation. Ultimately though, the antifibrotic effects conferred by compounds derived from TCM target critical signaling nodes such as TGF-β/Smad which play pivotal roles in EMT and ECM synthesis (Na et al., 2018) (Figures 2, 3). This provides a broad application prospect for the application of TCM in the treatment of renal fibrosis. Therefore, we provide an introduction to the key natural products in TCM that have had ameliorative effects on renal fibrosis (Supplementary Table S1) and review their associated mechanisms of action. Concurrently, given the prevalence, complexity, and consequences of renal fibrosis, this study aims to promote the development of TCM for treating renal fibrosis.

## 2 Single components of natural products used for renal fibrosis

Natural product constituents demonstrate notable efficacy in reversing or mitigating renal fibrosis through diverse molecular mechanisms. Based on a comprehensive literature review, this paper highlights five principal bioactive classes derived from TCM: glycosides, flavonoids, phenolic compounds, anthraquinones, and terpenoids. Each category exhibits distinct yet synergistic renoprotective effects, targeting key pathways implicated in fibrotic progression.

### 2.1 Glycosides

#### 2.1.1 Astragaloside IV

*Astragali radix* is deemed a safe herb and is frequently found in dietary supplements and health foods (Meifang et al., 2024). Glycosides are major constituents of *A. radix*. The glycosides identified include astragalosides I-VIII, acetylastragaloside, isoastragaloside I, isoastragaloside III, astramembrannin II, cycloastragenol, cyclosieversigenis, soyasaponin I, soyasapogenol B, and lupeol (Kathy et al., 2016). Among these, *Astragaloside IV* (AS-IV) was selected as a chemical marker in the Chinese pharmacopoeia for quality control purposes (Yiwei et al., 2023; Patrick Kwok-Kin et al., 2012). Moreover, it is also an important active constituent of Astragali radix, with multiple biological activities to ameliorate renal fibrosis, including the inhibition of oxidative stress and inflammation, as well as the modulation of autophagy.

Extant studies have found that AS-IV has protective effects on the kidneys and improves renal fibrosis by enhancing the expression of phosphorylated (p-) Smad3 C/p21 and nuclear factor (erythroid-derived 2)-like 2(Nrf2)/Heme oxygenase-1(HO-1), thereby inhibiting p-Smad 3L/plasminogen activator inhibitor (PAI)-1 and reducing ROS and α-SMA levels (Qin et al., 2023). And AS-IV mitigates Tacrolimus-induced chronic nephrotoxicity by enhancing the phosphorylation of p62, which subsequently promotes the nuclear translocation of Nrf2. This cascade effect ultimately alleviates the accumulation of ROS and attenuates renal fibrosis (Ping et al., 2021).

Notably, microRNAs (miRs) are short noncoding RNAs that negatively regulate gene expression posttranscriptionally. AS-IV can inhibit excessive mesangial cell proliferation and renal fibrosis via the modulation of the TGF-β1/Smad/miR-192 signaling pathway (Qian et al., 2019). In addition, AS-IV ameliorates renal function and renal fibrosis by inhibiting podocyte dedifferentiation and mesangial cell activation, which is induced by miR-21 (Xiaolei et al., 2018a).

In contrast, AS-IV inhibits inflammatory cell infiltration and inflammatory cytokine secretion (TNF-α, IL-6, and IL-1β) by inhibiting inflammation via toll-like receptor 4 (TLR4)/nuclear factor-κB (NF-κB), both *in vivo* and *in vitro* (Xiangjun et al., 2016; Jia-Long et al., 2022). Furthermore, AS-IV inhibits glucose-induced EMT of podocytes via the Sirt-NF-κB p65 axis (Xiaolei et al., 2018b). Besides, *in vivo* and *in vitro* experiments have revealed that AS-IV can also effectively ameliorate renal fibrosis by alleviating EMT procession. This may be due to the AS-IV-induced upregulation of the expression of ALDH2, which inhibits autophagy by regulating the AKT/mTOR pathway (Dong et al., 2023). In addition, the cGAS/STING signaling cascade represents a pivotal mechanism in mediating DNA-driven immune responses and inflammatory processes within inflammatory cells, including immune cells and injured renal parenchymal cells implicated in renal fibrosis (Yadan et al., 2024; Hui et al., 2024). These cells synthesize and release a plethora of profibrotic cytokines, such as TGF-β1, Wnt ligands, and angiotensin II. Subsequent activation of signaling pathways-including TGF-β, Wnt, renin-angiotensin-aldosterone system (RAAS), and Notch-elicits myofibroblast differentiation and accelerate ECM overproduction (Qian et al., 2022). Notably, in macrophages, this pathway is triggered during renal fibrogenesis by double-stranded DNA emanating from injured renal tubular epithelial cells (RTECs), thereby instigating a robust inflammatory cascade (Baihai et al., 2025). Experimental studies using cGAS- or STING-deficient murine models demonstrated marked attenuation of proinflammatory macrophage activation, myofibroblast accumulation, collagen deposition, and ECM synthesis following obstructive renal injury (Dong et al., 2023). Furthermore, chronic thermal stress was shown to amplify cGAS-STING pathway activity, concomitant with upregulated transcription of fibrosis-associated genes (e.g., collagen (COL) 1A1, α-SMA, and TGF-β) in renal tissues (Fumika et al., 2023).

#### 2.1.2 Ginsenoside Rg1

*Ginseng*, often referred to as the “king of herbs” is a valuable herb to remedy tissue growth related to fibrosis. Ginseng extract and its formulations can effectively inhibit the excessive deposition of ECM resulting from repeated injuries. Among its components, ginsenoside Rg1 is a prominent active ingredient in ginsenoside triol saponin, celebrated for its anti-inflammatory, anti-tumor, and antioxidant benefits.

Studies have found that ginsenoside Rg1 effectively lowers TGF-β1 activity and p-Smad levels. And it leads to significant inhibition of thrombospondin-1(TSP-1) expression, a cytokine known to promote TGF-β1 mRNA transcription and activation (Xie et al., 2008). Ginsenoside Rg1 also effectively inhibits decreases in α-SMA and E-calmodulin levels by suppressing the expression of p-ERK1/2, thereby leading to reduced COL Ⅰ and fibronectin levels in a dose-dependent manner, thus inhibiting EMT (Xi-Sheng et al., 2008). In addition, ginsenoside Rg1 protects against renal fibrosis by regulating the Klotho/TGF-β1/Smad signaling pathway (Sha-Sha et al., 2018).

Furthermore, study has demonstrated that ginsenoside Rg1 significantly improves lipid deposition, fibrosis, and ROS production by modulating MAPK and the downstream pathways in kidneys (Pengmin et al., 2023). Additionally, ginsenoside Rg1 exhibits nephroprotective effects by the upregulation of Nrf2-mediated HO-1 expression (Bin et al., 2019). Integrative informatics analysis identifies that ginsenoside Rg1 improves renal fibrosis through the regulation of autophagy (Yingying et al., 2024) and effectively increases superoxide dismutase (SOD) activity (Nan et al., 2014). Moreover, it reduces autophagy via the AMPK/mTOR (Nikolaos, 2020; Erine et al., 2021) and AKT/GSK3β/β-catenin pathways (Yimin et al., 2020).

#### 2.1.3 Salidroside

*Salidroside* (Sal) is an active compound derived from Rhodiola rosea L., which is a perennial alpine plant from the Crassulaceae family and is renowned for its unique medicinal properties that exhibit various pharmacological effects, including antifibrotic, anticancer, antidepressant, anti-inflammatory, antioxidant, antiulcer, and cardioprotective properties (Meihua et al., 2022).

Treatment with Sal can ameliorate tubular injury and deposition of the ECM components (including COLI and COLⅢ) as well as suppress EMT. Additionally, Sal also reduces the levels of serum biochemical markers (serum creatinine, blood urea nitrogen (BUN), and uric acid (Sixia et al., 2022) and decreases the release of inflammatory cytokines (IL-1β, IL-6, TNF-α). Treatment with Sal also significantly decreases the release of inflammatory cytokines and inhibits the TLR4/NF-κB and MAPK signaling pathways (Rui et al., 2019).

Further, the administration of Sal improves proteinuria, enhances nephrin and podocin expressions, and alleviates renal fibrosis and glomerulosclerosis primarily by inhibiting the β-catenin signaling pathway (Xinzhong et al., 2019). Furthermore, it decreases the mRNA and protein levels of Wnt1, Wnt3, TGF-β1, Axin-2, fibronectin, COLIII, p-Smad3, β-catenin, and the level and activity of cleaved caspase-3. Notably, Sal also reduces fasting blood glucose levels and renal ROS, while it increases SOD and glutathione levels. This has been found to ultimately result in the protection of rat kidneys from injury and fibrosis. These protective effects are achieved through GS3Kβ-mediated inhibition of Wnt1/Wnt3a β-catenin, combined with hypoglycemic and antioxidant effects (Ali and Mohammad, 2020).

Briefly, of the three glycoside natural products described above, AS-IV can improve renal fibrosis through multiple pathways. In addition, ginsenoside Rg1 has the potential to ameliorate renal fibrosis; however, the slow growth cycle, low seed yield, and prolonged generation time of ginseng have led to a disparity between the demand for and supply of ginsenosides. Additionally, we found that Sal has protective effects mainly in cardiovascular and neurodegenerative diseases. Therefore, the mechanism by which Sal ameliorates renal fibrosis needs to be further explored.

### 2.2 Flavonoids

#### 2.2.1 Quercetin

*Quercetin*, the predominant flavonoid found in *Flos Sophorae Immaturus*, has been extensively researched for its protective effects on the kidneys. Studies have demonstrated that the use of quercetin leads to a reduction in fibrosis, apoptosis, nephrotoxicity, and inflammation associated with various kidney diseases (Azar et al., 2021; Priyanka et al., 2021).

Further, research indicates that quercetin mitigates endoplasmic reticulum stress by promoting Sirt1-mediated deacetylation of Nrf2, NF-κB p65, eIF2α, and xbp-1 (Ghedeir et al., 2021). Moreover, quercetin prevents CKD by regulating inflammation and oxidative stress (Wahyu et al., 2022). It also alleviates fibrosis and the accumulation of inflammation-related proteins through the IL33/ST2 signaling pathway (Hsin-Yuan et al., 2022). In human kidney-2 (HK-2) and the normal rat renal tubular epithelial cell line, quercetin counteracts EMT and renal fibrosis by activating mTORC1/p70S6K (Qian et al., 2015). Furthermore, it hampers Hedgehog signaling activation, thereby reducing obstructive renal fibrosis and EMT by downregulating the Amphiregulin/epidermal growth factor receptor pathway (Qi et al., 2022).

Cao et al. found that quercetin demonstrates the ability to attenuate TGF-β-induced fibrosis by inhibiting miR-21 and increase the levels of PTEN as well as the tissue inhibitor of metalloproteinase 3 (Yaochen et al., 2018). According to Tu et al. (Haitao et al., 2021), quercetin effectively treats renal fibrosis by modulating PIK3R1 and inhibiting the PI3K/Akt pathway, which helps alleviate fibrosis and apoptosis in renal tissues. Furthermore, it also mitigates renal fibrosis by decreasing the senescence of RTECs via the Sirt1/PINK1/mitochondrial autophagy pathway (Tao et al., 2020).

#### 2.2.2 Baicalin

*Baicalin* is a key ingredient found in *Scutellaria*, one of the substances used in TCM in China (Yongqiang et al., 2023). Recent studies have revealed the significant anti-fibrotic capabilities of baicalin. The protective effect of baicalin on fibrosis is largely attributed to its ability to block TGF-β and inflammatory reaction (Ning et al., 2021). Specifically, baicalin treatment can alleviate renal interstitial fibrosis, and the mechanism may be related to the inhibition of TGF-β1 expression by inhibiting the Notch1 signaling pathway (Qin et al., 2017; Hui W. et al., 2022; Yi et al., 2017; Yu-Jie et al., 2016).

In addition, Baicalin exerts antifibrotic effects in the kidney by modulating signal transducer and activator of transcription 6 (STAT6) signaling. Mechanistically, STAT6 mediates transcriptional repression of peroxisome proliferator-activated receptor α and its downstream fatty acid oxidation-related genes, culminating in lipid accumulation within RTECs—a metabolic perturbation that drives fibrotic progression (Youjing et al., 2023). Concurrently, STAT6 activation facilitates macrophage-to-myofibroblast trans-differentiation and amplifies M2 macrophage polarization, a subset known to secrete profibrotic mediators that exacerbate renal fibrogenesis (Tianhui et al., 2023). This process is further reinforced by glycoprotein non-metastatic melanoma protein B, which synergizes with the IL-4-STAT6 axis to potentiate M2 polarization (Yahong et al., 2020; Letian et al., 2017). Under hyperglycemic conditions, mesangial cells exhibit elevated expression of fibrogenic markers, including TGF-β, fibronectin, and collagen.

#### 2.2.3 Puerarin

*Puerarin* is an isoflavonoid isolated from the root of the plant *Pueraia* and has been widely used in traditional Chinese herbal medicine for the treatment of various renal diseases, such as renal fibrosis, diabetic kidney disease, kidney stone, acute kidney injury (AKI) and CKD (Xueling et al., 2024; Zujian et al., 2024; Yuexian et al., 2024). Consequently, puerarin has garnered significant attention for its therapeutic potential.

A recent study revealed that puerarin alleviates unilateral ureteral obstruction (UUO)-induced inflammation and fibrosis by regulating the NF-κB p65/STAT3 and TGFβ1/Smads signaling pathways (Jingyu et al., 2021). Furthermore, puerarin treatment ameliorates renal fibrosis by inhibiting epithelial cell apoptosis through the MAPK signaling pathway (Xiangjun et al., 2017). Additionally, puerarin alleviates oxidative stress and ferroptosis during renal fibrosis induced by ischemia/reperfusion injury via TLR4/(NADPH) oxidase (Nox) four pathway in rats (Jun et al., 2023). Moreover, research using *in silico* prediction and experimental validation identified puerarin’s protective mechanism against excessive ECM accumulation through inhibiting ferroptosis (Biyu et al., 2023).

Overall, flavonoids are potent antioxidants with the potential to attenuate tissue damage. Considering the multicomponent nature of natural products with diverse effects and characteristics, there remains a need for more in-depth research on its pharmacological effects and related mechanisms in modern medicine.

### 2.3 Phenols

#### 2.3.1 Curcumin

*Curcumin*, a polyphenol pigment derived from *turmeric*, is extensively used in the food industry to enhance the color and flavor of consumable products, such as pasta, meat, and beverages. Beyond its use as a food coloring, curcumin provides numerous biological and pharmacological benefits, potentially offering anti-fibrosis, anti-cancer, anti-thrombotic, anti-heart failure, anti-inflammatory, and blood pressure-lowering properties, which makes it valuable in medical context (Aliabbas et al., 2020; Mahvash et al., 2023). Therefore, it is widely utilized.

Prior studies have proved that curcumin can ameliorate renal fibrosis by inhibiting the TGF-β1/Smad signaling pathway (Chen et al., 2021; Chadanat and Visith, 2024), and the mechanism blocks its profibrotic actions on renal fibroblasts through the downregulation of TβRII, partial inhibition of c-Jun activity (Jens et al., 2004), and through reversing ADAMTS18 gene methylation (Ben et al., 2023). Furthermore, curcumin ameliorates renal fibrosis by inhibiting local fibroblast proliferation and ECM deposition, upregulating the expression of peroxisome proliferator-activated receptor gamma (PPAR-γ), and downregulating the expression of p-Smad2/3 (Xiangjun et al., 2014). In addition, it prevents fibroblast activation through the mitigation of intracellular free radicals and TGF-β secretion (Chadanat and Visith, 2024).

Curcumin can also alleviate oxidative stress and inflammation (Cecilia Gabriela et al., 2024). Specifically, curcumin has been found to be able to scavenge—in a concentration-dependent manner—superoxide anion, hydroxyl radical, peroxyl radical, singlet oxygen, peroxynitrite anion, hypochlorous acid, and hydrogen peroxide (Joyce et al., 2015). In 5/6 nephrectomized rats, curcumin has been found to induce Nrf2 nuclear translocation; prevent glomerular hypertension, hyperfiltration, and oxidant stress; decrease antioxidant enzymes; and reverse glomerular hemodynamic alterations (Edilia et al., 2012). It may also be significant in cellular antioxidant defense through the activation of the Nrf2-keap1 pathway (Xiuli et al., 2015; Vivian et al., 2012). Moreover, it has anti-fibrosis effects via the inhibition of the activation of the TLR4-NF-κB signal pathway (Zhaohui et al., 2020) and reversing caveolin-1 Tyr14 phosphorylation (Li-Na et al., 2014).

#### 2.3.2 Epigallocatechin-3--gallate

Polyphenols derived from green tea have been reported to possess a wide range of profound functions (Wang et al., 2019). *Epigallocatechin-3-O-gallate* (EGCG) is the most active and abundant polyphenol in *green tea* (Avila-Carrasco et al., 2021). And EGCG has been reported in several renal disease models, such as AKI, cisplatin-induced nephrotoxicity, obstructive nephropathy, glomerulonephritis, lupus nephritis, diabetic nephropathy, and high-fat diet-induced kidney injury (Luo et al., 2020).

In an UUO mice model, EGCG was found to attenuate renal fibrosis by inhibiting the accumulation of ECM and EMT, and this renoprotective effect might be associated with its effect of the alleviation of inflammatory responses and TGF-β/Smad signaling pathway inhibition (Wang et al., 2015a). Additionally, EGCG was found to ameliorate the CdCl2-induced renal injury and fibrosis, inhibit the level of oxidative stress, normalize renal enzymatic antioxidant status and E-cadherin level, as well as attenuate the over-generation of vimentin and α-SMA (Chen et al., 2016). Similarly, through its antioxidant and epigenetic modulation capacities, EGCG has protective effects against arsenic-induced cytotoxicity and fibrogenic changes in kidney epithelial cells (Iheanacho et al., 2024).

Luo et al. found that EGCG could lower MDA levels, reduce the numbers of infiltrated macrophages and T cells, and induce apoptosis (Luo et al., 2020). Specifically, it can reduce B-cell lymphoma-2 (Bcl-2) and increase Bax and cleaved caspase 3 (Wang et al., 2015b).

#### 2.3.3 Resveratrol

*Resveratrol* is a versatile phenolic compound commonly found in various plants-particularly knotweed-as well as in *mulberry, peanuts, buyer’s twine, and Korean acacia* (Uddin et al., 2021). *In vitro* and *in vivo* studies have confirmed the health benefits of resveratrol in kidney diseases (Den Hartogh and Tsiani, 2019). Resveratrol, an SIRT1 activator, effectively prevented ROS generation, production of ECM proteins, mitochondrial damage, and senescence (Dong Ryeol et al., 2019).

Studies have revealed that resveratrol inhibits renal interstitial fibrosis by regulating the AMPK/NOX4/ROS pathway (Ting et al., 2016). Furthermore, AKT/FOXO3a signal pathway mediates the protective mechanism of resveratrol on renal interstitial fibrosis and oxidative stress (Rongrong and Qu, 2022). Specifically, resveratrol decreased the levels of MDA and 8-OHdG, and increased the level of SOD, which protects cells against ROS damage (Jin et al., 2013). Sener Göksel et al. found that resveratrol exerts renoprotective effects via its radical scavenging and antioxidant activities, which appear to involve the inhibition of tissue neutrophil infiltration (Felix et al., 2024). Additionally, resveratrol has a protective effect on EMT by suppressing oxidative stress and a possible involvement of TGF-β/Smad signaling pathway (Beshay et al., 2020).

Resveratrol can act on the TGF-β pathway through multiple targets and subsequently attenuate renal injury and fibrosis. For example, resveratrol can inhibit matrix metalloproteinase (MMP) 7 (Zhou et al., 2015). Huang et al. demonstrated that SIRT1 can bind to Smad3 via co-immunoprecipitation. Resveratrol treatment enhanced this binding and reduced the acetylation levels of Smad3. Simultaneously, resveratrol inhibited the transcription activity of Smad3 (Xin-Zhong et al., 2013).

Another important property of resveratrol is its anti-aging resistance. Senescence contributes to tubular epithelial cell damage (Deping et al., 2024). The serum levels of advanced glycation end-products and renal function markers BUN, creatinine, and cystatin C in mice have been found to significantly increase after the administration of D-galactose, and this outcome could be significantly reversed by treatment with resveratrol (Kuo-Cheng et al., 2023). Furthermore, resveratrol treatment alleviated age-related EMT in aging kidneys, which was accompanied by the activation of AMPK-mTOR signaling (Dan et al., 2017). Moreover, it reinforces the therapeutic effect of mesenchymal stem cell-derived exosomes against renal fibrosis by suppressing EMT (Fuhe et al., 2024).

#### 2.3.4 Salvianolic acid B

*Salvianolic acid B* (SalB) is the principle water-soluble active component of *Salvia miltiorrhiza*, derived from three salvianin molecules and one caffeic acid molecule.

Recent findings have revealed that SalB protects against renal fibrosis by reversing EMT. Wang et al. discovered that SalB activates the TGF-β1/Smads signaling pathway both *in vivo* and *in vitro*, which helps to prevent EMT (Wang et al., 2010). In a study by He et al., SalB was found to improve renal function and lower the levels of fibronectin, α-SMA, and TGF-β. In addition, SalB mitigated EMT related to renal fibrosis via SIRT1-mediated autophagy (He et al., 2020). Furthermore, SalB regulates the expression of miR-106b-25 and preserves the epithelial traits of HK-2 cells by lowering α-SMA levels and increasing the E-cadherin level (Tang et al., 2014).

In order to investigate the effect of SalB on renal tubulointerstitial fibrosis and explore the potential mechanisms, Lin et al. used two models of renal fibrosis-UUO and aristolochic acid nephropathy. The results revealed that it significantly increased the levels of Scr and BUN, suppressed the expression of fibronectin and α-SMA, increased PTEN, and decreased p-Akt, which correlated with the downregulation of the enhancer of zeste homolog-2 (EZH2) and histone H3 lysine 27 trimethylation (H3K27me3) (Lin et al., 2023). EZH2 is a methyltransferase that induces H3k27me3 and regulates gene transcription in fibrogenesis (Diana Valeria, 2020). Additionally, SalB alleviates renal fibrosis by modulating platelet-derived growth factor (PDGF)-C/PDGFR-α pathway (Yao et al., 2022) and heparinase/syndecan-1 axis (Hu et al., 2020).

Phenols, as widespread secondary metabolites in plants, have significant biological activities, but their efficacy is often limited by self-deficiency such as low absorption of curcumin, poor stability of resveratrol, and easy and rapid degradation of SalB, which leads to insufficient bioavailability. However, there have been many successful cases demonstrating the critical role of nanomaterials: for example, natural ursolic acid-based carriers can deliver resveratrol in a targeted manner to repair renal injury (Nie et al., 2023). Methoxypolyethylene glycol-chitosan to enhance the oral delivery and kidney-targeted distribution of SalB (Zhang et al., 2023). These advances suggest that the inherent defects of the molecule can be effectively circumvented by tailor-designed nano-delivery systems, thereby substantially enhancing its bioavailability. Thus, phenolic compounds are expected to be important new strategies for renoprotection and antifibrosis.

### 2.4 Anthraquinones

#### 2.4.1 Rhein

*Rhein* is identified as an anthraquinones and serves as a vital ingredient in several types of TCM, including *Rheum palmatum L*., Polygonum multiflorum, and aloe vera (Cheng et al., 2021; Zhu et al., 2022).

Recent studies have emphasized rhein’s ability to inhibit mesangial cell proliferation, ECM production, and TGF-β1 expression in human renal cells. Additionally, rhein alleviates desmoplastic anemia and EMT by influencing the Ras-related C3 botulinum toxin substrate 1/NOX1/β-catenin signaling pathway (Xiong et al., 2023). In a UUO model, rhein was found to reduce renal interstitial fibrosis by modulating the Sonic hedgehog-Glioma-related cancer gene homologous proteins 1-Snail signaling pathway (Luo et al., 2022). In addition, rhein reduces renal fibrosis by promoting Cpt1a-mediated fatty acid oxidation through the Sirt1/STAT3/twist1 pathway (Chen Y. et al., 2019; Song et al., 2022), thereby leading to an improvement in renal function and reducing interstitial damage and collagen fiber accumulation by activating the Sirt3/FOXO3a pathway (Wu et al., 2020). Based on the integrated network pharmacology and the construction of the rhein-target-metabolic enzyme-metabolite network, Xiao et al. found that rhein played an antifibrotic role through the PPAR-α-CPT1A-l-palmitoyl-carnitine axis (Qiming et al., 2022).

Interestingly, rhein reversal of DNA hypermethylation-associated Klotho suppression ameliorates renal fibrosis (Zhang et al., 2016), and it also provides renal protection through the regulation of DNA methyltransferases expression and methylation at the Klotho promoter (Zhang et al., 2017). Furthermore, treatment with rhein has effectively reversed alterations in Klotho and TLR4, thereby reducing inflammatory responses (Bi et al., 2018).

#### 2.4.2 Emodin

*Rheum palmatum* is a commonly used herb in TCM for the treatment of AKI. The main active component of rhubarb is emodin, which was first recorded in Shennong’s Classic of Materia Medica. Emodin has been found to be effective against renal fibrosis and has been widely studied for its effects on kidney diseases (Liu et al., 2022).

Emodin reduces proteinuria and alleviates renal fibrosis. The potential mechanisms by which emodin exerts its renoprotective effects are through suppressing cell apoptosis and enhancing the autophagy of podocytes via the AMPK/mTOR signaling pathway in the kidney (Liu H. et al., 2021). Additionally, studies have demonstrated that emodin ameliorates renal injury and fibrosis via regulating the miR-490-3p/high migration protein A2 axis (Wang L. et al., 2023), retarding renal fibrosis through regulating HGF and TGF-β/Smad signaling pathway (Ma et al., 2018), hindering EMT via regulation of bone morphogenetic protein-7/TGF-β1 in renal fibrosis (Liu W. et al., 2021), suppressing IL1β-induced mesangial cells proliferation and ECM production via inhibiting p38 MAPK (Wang et al., 2007), and improving renal fibrosis in CKD by regulating mitochondrial homeostasis through the mediation of peroxisome proliferator-activated receptor-gamma coactivator-1 α (Feng L. et al., 2024).

Interestingly, deoxycholic acid-chitosan coated liposomes combined with *in situ* colonic gel enhances renal fibrosis therapy of emodin (Xu et al., 2022). However, the poor solubility, limited colonic irrigation retention time, and inadequate colon adhesion of emodin hinder its clinical application. Consequently, Lu et al. combined emodin with the nanoparticles to prepare an emodin-nanoparticle system (emodin-NP) and studied their efficacy in delaying CKD progression. The emodin-NP alleviates kidney dysfunction and tubulointerstitial fibrosis by mediation through the modification of gut microbiota disorders (Lu et al., 2020).

In summary, there remains a lack of clinically relevant data on rhein and emodin, thereby necessitating further exploration into its clinical efficacy as well as appropriate dosing and treatment regimens. Simultaneously, future research should focus on the application of network pharmacology and bioinformatics.

### 2.5 Terpenoids

#### 2.5.1 Poricoic acid A

*Poricoic acid A* (PAA) isolated from *Poria cocos* is a potent anti-fibrotic agent. Studies have suggested that the suppression of TGF-β1-induced renal fibroblast ECM accumulation, fibrosis formation, and proliferation by PAA is mediated via the inhibition of the PDGF-C, Smad3, and MAPK pathways (Chen et al., 2020). In the UUO mice model, PAA reduced the activity of the Wnt/β-catenin signaling pathway by enhancing the expression of tryptophan hydroxylase-1 and also inhibited renal cell injury and fibroblast activation, thereby exerting an anti-fibrosis effect (Dan-Qian et al., 2020). Molecular docking analysis revealed that there may be a potential interaction between SIRT3 and PAA. Then, in both *in vivo* and *in vitro* models, PAA was found to attenuate renal fibroblast activation and interstitial fibrosis by upregulating SIRT3 and inducing β-catenin K49 deacetylation (Chen et al., 2023). Interestingly, PAA enhances melatonin inhibition of AKI-to-CKD transition by regulating Gas6/Axl NFκB/Nrf2 axis (Chen et al., 2019b). Combined melatonin and PAA inhibit renal fibrosis through modulating the interaction of Smad3 and the β-catenin pathway in the AKI-to-CKD continuum (Chen et al., 2019c).

#### 2.5.2 Tanshinone IIA

*Tanshinone IIA* is a diterpene extracted from *S. miltiorrhiza*, a popular and safe herb medicine that has been widely used in China.

Tanshinone IIA was used to target oxidative stress and inflammation for the improvement of fibrosis (Lu et al., 2022; Jiang et al., 2016). Mechanistically, tanshinone IIA ameliorates renal fibrosis by suppressing the TGF-β/TSP-1 (Fan et al., 2024), regulating NRF2/NLRP3 (Zhang et al., 2024), NF-κB (Wang D. et al., 2015), miR-34-5p/Notch1 axis (Zhang and Yang, 2022), and TGF-β/Smad signaling pathways. Moreover, tanshinone IIA ameliorates EMT via the Akt/mTOR/p70S6K (Jiang et al., 2019), Vitamin D receptor/Wnt/β-catenin signaling pathways (Zeng and Bao, 2021; Zeng et al., 2024; Cao et al., 2017). In addition, the kidney protective and antifibrotic effect of tanshinone IIA was likely attributable to an early inhibition of renal recruitment of fibrocytes positive for both CD45 and COL I (Jiang et al., 2015). Meanwhile, tanshinone IIA may be associated with reduced ER stress via attenuating PERK signaling activities (Xu et al., 2020).

#### 2.5.3 Bixin

*Bixin*, a carotenoid derived from the seeds of *Bixa orellana*, belongs to a distinctive subclass of terpenoids and exhibits multifaceted pharmacological properties, including antifibrotic, anti-inflammatory, and antioxidant activities (Jianzhong et al., 2020a; C et al., 2001; Shasha et al., 2016). Mechanistically, bixin suppresses the TLR4/MyD88/NF-κB and TGF-β1/Smad3 signaling axes, attenuating the secretion of proinflammatory cytokines such as TNF-α and IL-1β (Jie-Qiong et al., 2020; Jianzhong et al., 2020b). Additionally, it enhances proteasomal degradation of STAT6, thereby mitigating renal interstitial fibrosis (Jianzhong et al., 2020a). Bixin further demonstrates antioxidative efficacy by upregulating endogenous defense systems, including SOD, catalase, glutathione peroxidase, PPAR-γ, NAD(P)H quinone dehydrogenase 1, HO-1, and Nrf2. Concurrently, it downregulates profibrotic mediators such as MMP9, TGF-β1, and fibronectin, collectively ameliorating oxidative stress and halting fibrotic progression in renal tissues (Shasha et al., 2016; Jie-Qiong et al., 2020; Jianzhong et al., 2020b; Hong et al., 2018).

In addition to those previously mentioned (Table 1), several other herbs such as *likeanthocyanins* (Li et al., 2022)*, mangiferin* (Lum et al., 2022)*, coffee leaf tea extracts* (Zhou et al., 2023)*, Lycium barbarum* (Liu et al., 2024; Lu et al., 2019)*, dihydromyricetin* (Wen et al., 2024; Liu et al., 2019)*, berberine* (Ahmedy et al., 2022; Yang et al., 2017; Hassanein et al., 2022)*, licorice* (Liao et al., 2020; Li et al., 2010)*, and mulberry leaf* (Wu, 2019; Hung et al., 2023; Ji et al., 2019) have been found to enhance renal fibrosis. However, the current studies on this are few and the mechanisms need to be further confirmed; additional research will likely provide new ideas for improving renal fibrosis. Overall, the protective effects of natural products for kidney health present a valuable area for further in-depth exploration.

**TABLE 1:** Description of natural products used in the treatment of renal fibrosis.

Categories	Monomers	Herbal sources	Structure*	Molecular mechanisms	References
Glycosides	Astragaloside IV	Astragali radix	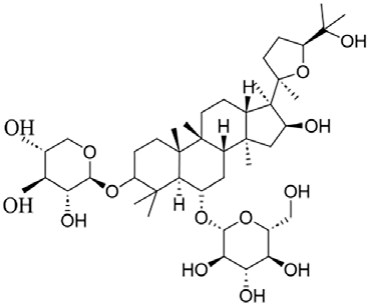	Anti-oxidant, anti-inflammatory, anti-fibrotic	Yiwei et al. (2023), Patrick Kwok-Kin et al. (2012)
	Ginsenoside Rg1	Ginseng	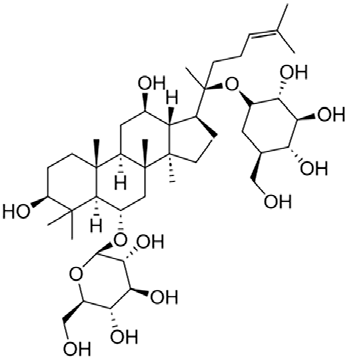	Anti-inflammatory, anti-tumor, anti-oxidant	Xie et al. (2008)
	Salidroside	Rhodiola rosea L	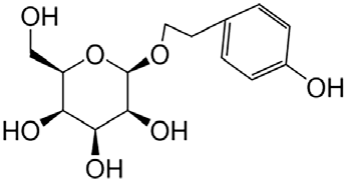	Anti-fibrotic, anti-cancer, anti-depressant, anti-inflammatory, anti-oxidant, anti-ulcer and cardioprotective	Meihua et al. (2022)
Flavonoids	Quercetin	Flos Sophorae Immaturus	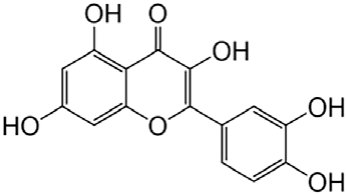	Anti-oxidant, anti-inflammatory, anti-apoptotic and anti-fibrotic	Azar et al. (2021), Priyanka et al. (2021)
	Baicalin	Scutellaria	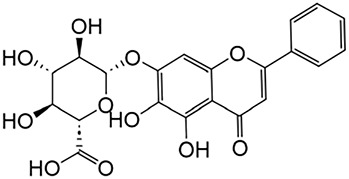	Anti-oxidant, anti-inflammatory, anti-infectious and anti-tumor	Yongqiang et al. (2023)
	Puerarin	Pueraia	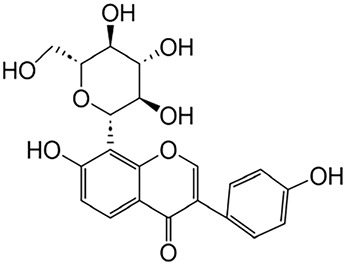	Anti-fibrotic, antioxidant, anti-inflammatory and immunomodulator	Jingyu et al. (2021)
Phenols	Curcumin	turmeric	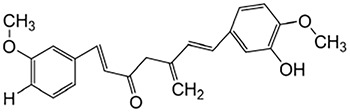	Anti-cancer, anti-thrombotic, anti-heart failure, inhibits inflammatory response and lowers blood pressure	Chen et al. (2021), Chadanat and Visith (2024)
	Epigallocatechin-3--gallate	Green tea	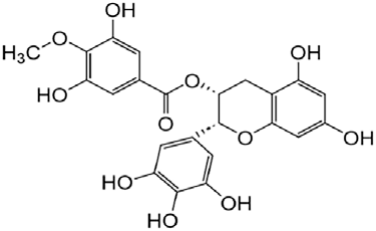	Anti-oxidant, NO-scavenging, anti-inflammatory, anti-arthritic and apoptosis	Luo et al. (2020)
	Resveratrol	Knotweed	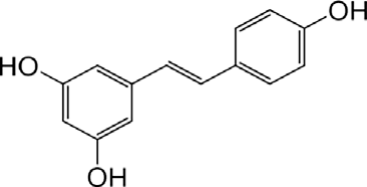	Regulating blood lipid levels, preventing LDL oxidation, anti-platelet aggregation, and anti-aging	Dong Ryeol et al. (2019)
	Salvianolic acid B	Slaviae miltiorrhizae	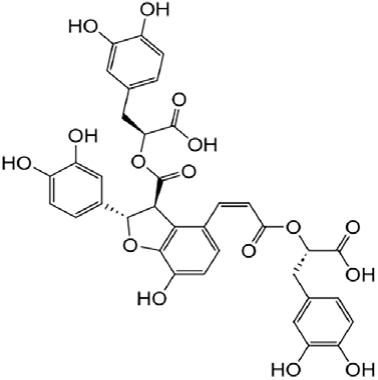	anti-inflammatory, anti-oxidant,and anti-apoptotic	Wang et al. (2010)
Anthraquinones	Rhein	Rheum palmatum L	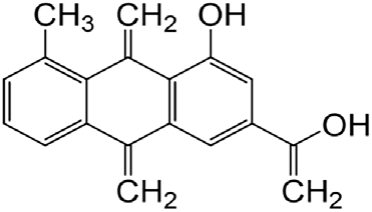	Anti-tumor, anti-inflammatory, anti-bacterial	Nie et al. (2023), Zhang et al. (2023)
	Emodin	Rheum palmatum L	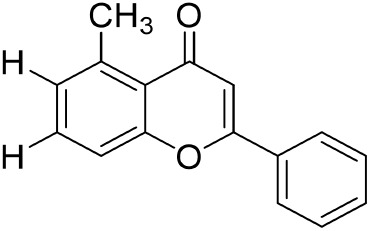	Anti-fibrotic anti-inflammatory	Zhang et al. (2017)
Terpenoids	Poricoic acid A	Wolfiporia cocos	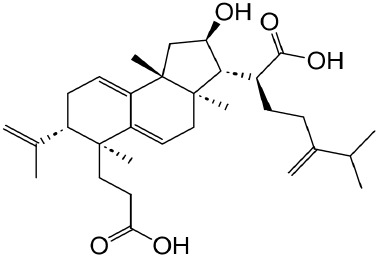	Anti-fibrotic	Xu et al. (2022)
	Tanshinone IIA	Salvia miltiorrhiza	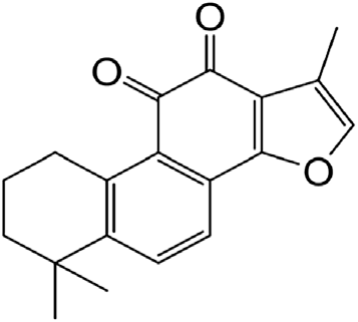	Anti-fibrotic	Chen et al. (2019b), Chen et al. (2019c)
	Bixin	Bixa orellana	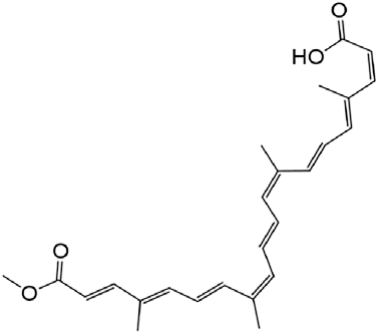	anti-fibrotic, anti-inflammatory, and antioxidant	Jiang et al. (2015), Xu et al. (2020), Jianzhong et al. (2020a)

*Represents the structures in this review were made by ChemDraw.

## 3 Combined application of TCM

In recent years, natural products have garnered significant attention as therapeutic agents for renal fibrosis. While single-herb interventions remain foundational in TCM research, the unique advantage of TCM lies in the synergistic integration of multiple bioactive compounds within formulated preparations. These formulations, meticulously designed to amplify therapeutic efficacy, represent the cornerstone of TCM’s holistic approach.

One innovative strategy involves the co-delivery of total rhubarb anthraquinone (TRA) and total astragalus saponin (TAS) via a self-nanoemulsifying drug delivery system (SNEDDS), optimized through ternary phase diagrams to enhance oral bioavailability. To address stability and drug-loading challenges, TRA/TAS-loaded SNEDDS were solidified into pellets using fluid-bed coating. Further refinement incorporated astragalus polysaccharides (APS) into colonic site-specific pellets through sustained-release and enteric coatings. Encapsulating TRA/TAS and APS pellets at a 1:2 mass ratio in hard capsules demonstrated spatiotemporal payload release via CHP Type I dissolution testing. In UUO rat models, this combined pellet system (CPs) attenuated renal histopathological damage, suppressed collagen deposition, and reduced proinflammatory cytokines. Notably, CPs restored gut microbiota dysbiosis and preserved intestinal barrier integrity, underscoring their multi-target therapeutic potential (Qibin et al., 2024).

Mechanistic studies reveal that classic TCM formulations exert antifibrotic effects through diverse pathways. The Astragali radix and Angelicae sinensis radix decoction modulates plasminogen activator/PAI and MMP/TIMP imbalances to mitigate ECM accumulation (Liqiang et al., 2012). Similarly, Huangqi-Danshen decoction targets stearoyl-CoA desaturase 1 to inhibit cGAS-STING signaling, disrupting fibrotic cascades (Dong et al., 2023; Hong et al., 2018; Yu et al., 2025). Synergistic combinations, such as AS-IV with ginsenoside Rg1, alleviate oxidative stress and TGF-β/Smad3-driven fibrogenesis (Erine et al., 2021). Fushengong Decoction, anchored by Astragalus, regulates the PTEN/PI3K/AKT/NF-κB axis to preserve renal function, with PTEN’s phosphatase activity counteracting oncogenic PI3K/AKT/mTOR signaling (Virginia et al., 2019; Changlong et al., 2021). Bioinformatic analyses further implicate PTEN/TGF-β crosstalk in renal fibrosis progression, influencing cell migration and motility (Yongchao et al., 2019). The Rhubarb-Astragalus Capsule mitigates apoptosis via TGF-β1/p38 MAPK inhibition in UUO models (Xian et al., 2020), while Perindopril Erbumine combined with Huangqi-Danshen Decoction attenuates adenine-induced CKD through Sirtuin3-mediated mitochondrial dynamics (Xian et al., 2022). Intriguingly, Shenqi Detoxification Granule suppresses EMT by modulating TGF-β1/Smad/ILK signaling alongside P311, a conserved profibrotic protein (Pingping et al., 2018).

Classic polyherbal formulations exemplify TCM’s multi-component synergy. The Shenkang Injection, comprising Astragalus, Rhubarb, Safflower, and Sage, attenuates fibrosis via dual targeting of IκB/NF-κB and Keap1/Nrf2 pathways, enhanced by anthraquinones like rhein and emodin (Fu et al., 2019; Liang-Pu et al., 2022). Liuwei Dihuang Pill, integrating Rehmannia, Coptis, and Cornu Cervi Pantotrichum, inhibits TGF-β1/MAPK signaling to reduce inflammation and interstitial fibrosis (Zhu et al., 2024). The Shen-Qi-Huo-Xue formula, validated pharmacopeial herbs including Pseudostellaria heterophylla and Salvia miltiorrhiza, restores Hypoxia-inducible factor (HIF)-1α/HIF-2α homeostasis to counteract tubular ferroptosis and EMT in diabetic nephropathy (Ronglu et al., 2025).

In summary, these studies elucidate the mechanistic and practical paradigms of TCM’s combinatorial strategies, encompassing herbal synergies, chemical drug integrations, and advanced delivery systems. By targeting multifaceted pathways—from ECM regulation to mitochondrial dynamics and microbiota modulation—these approaches offer transformative insights for both preclinical research and clinical translation in renal fibrosis management.

## 4 Discussion

### 4.1 Challenges in natural product utilization

#### 4.1.1 Phytochemical complexity and mechanistic ambiguity

Natural products are inherently complex, comprising hundreds of bioactive constituents with unpredictable synergistic or antagonistic interactions. This heterogeneity complicates the elucidation of pharmacokinetic and pharmacodynamic profiles, particularly in compounded preparations, thereby hindering standardization and clinical validation.

#### 4.1.2 Geographical variability and quality control

The chemical composition of medicinal plants is significantly influenced by environmental factors (e.g., soil composition, climate, and cultivation practices), and geographic differences lead to variations in plant chemical composition, which in turn affects quality control and production stability. For example, Klein-Junior et al. (2021) noted that phenotypic differences in plants are due to geographic variation, which poses a challenge to batch-to-batch stability, and thus affects consistency in large-scale production. Therefore, modern quality control methods must be employed to counteract the effects of this geographic variability on the consistency of drug quality.

#### 4.1.3 Optimization of delivery modalities

Route of administration has a crucial impact on drug efficacy. Different modes of administration such as oral administration, intravenous injection and topical application affect the bioavailability and therapeutic efficacy of drugs.Wang H. et al. (2023) showed that geographical variability of herbs not only affects their chemical composition, but also has a significant impact on drug absorption and bioavailability. For example, the absorption of certain herbal components may be unstable when administered locally, and thus the delivery method needs to be optimized for the characteristics of different herbs to improve the therapeutic efficacy. In this way, the therapeutic efficacy of drugs can be significantly improved by rationally selecting the route of administration.

#### 4.1.4 Drug development and safety profiling barriers

Isolation of high-purity bioactive compounds from complex herbal matrices remains technically challenging, particularly for low-abundance metabolites. Although natural products are perceived as safer than synthetic drugs, their toxicological profiles-especially organ-specific effects-are undercharacterized. For instance, celastrol demonstrates antifibrotic efficacy in pulmonary models (Divya et al., 2018; Divya et al., 2016), yet its renal implications remain unexplored (Zhou et al., 2022), necessitating targeted toxicokinetic evaluations.

#### 4.1.5 Limitations in clinical evidence and translational relevance

Current clinical research on natural products is marred by methodological deficiencies, including small cohorts, inadequate controls, and non-standardized endpoints. Preclinical findings from animal or *in vitro* models often fail to replicate human pathophysiology due to interspecies metabolic and immunological disparities. Human trials must account for variables such as sex-specific pharmacokinetics, age-related renal decline, and gut microbiota diversity, which modulate therapeutic response.

### 4.2 Future directions and strategic innovations

To overcome these challenges, interdisciplinary collaboration integrating medicinal chemistry, pharmacology, and chemical biology is imperative. Emerging technologies—such as artificial intelligence-driven drug design, bioinformatics, and nanobiotechnology—offer novel avenues to enhance compound bioactivity and reduce toxicity. Metabolomics and network pharmacology can elucidate drug mechanisms and identify disease-specific molecular targets (Luo et al., 2024; Li et al., 2023). Additionally, semi-synthetic and biosynthetic approaches may address sourcing limitations while improving solubility and bioavailability.

Despite inherent challenges, natural products hold transformative potential for CKD and renal fibrosis management. By harmonizing TCM’s holistic principles with modern precision medicine, and leveraging technological advancements, future research can bridge the gap between empirical tradition and evidence-based therapeutics.

## Glossary

α-SMA: α-smooth muscle actin
AKI: acute kidney injury
APS: astragalus polysaccharides
AS-IV: Astragaloside IV
BUN: blood urea nitrogen
cGAS: cyclic GMP-AMP synthase
CKD: chronic kidney diseases
CPs: combined pellet system
ECM: extracellular matrix
EGCG: Epigallocatechin-3-O-gallate
NP: nanoparticle
EMT: epithelial-mesenchymal transition
EZH2: enhancer of zeste homolog-2
H3K27me3: histone H3 lysine 27 trimethylation
HO-1: Heme oxygenase-1
IL-1β: interleukin-1β
miRs: microRNAs
MMP7: matrix metalloproteinase7
PAA: Poricoic acid A
PAI: plasminogen activator inhibitor
PDGF: platelet-derived growth factor
RAAS: renin-angiotensin-aldosterone system
RTECs: renal tubular epithelial cells
Sal: Salidroside
SalB: Salvianolic acid B
SNEDDS: self-nanoemulsifying drug delivery system
SOD: superoxide dismutase
STAT6: signal transducer and activator of transcription 6
STING: stimulator of interferon genes
TAS: total astragalus saponin
TCM: Traditional Chinese Medicine
TRA: total rhubarb anthraquinone
TSP-1: thrombospondin-1
UUO: unilateral ureteral obstruction
